# Diagnostic application of intraoral ultrasonography to assess furcation involvement in mandibular first molars

**DOI:** 10.1259/dmfr.20230027

**Published:** 2023-05-12

**Authors:** Ray Tanaka, Katherine Lau, Andy WK Yeung, Wai Keung Leung, Takafumi Hayashi, Michael M. Bornstein, Maurizio S. Tonetti, George Pelekos

**Affiliations:** 1 Oral and Maxillofacial Radiology, Applied Oral Sciences & Community Dental Care, Faculty of Dentistry, The University of Hong Kong, Hong Kong, China; 2 Periodontology & Implant Dentistry, Faculty of Dentistry, The University of Hong Kong, Hong Kong, China; 3 Division of Oral and Maxillofacial Radiology, Niigata University Graduate School of Medical and Dental Sciences, Niigata University, Niigata, Japan; 4 Department of Oral Health & Medicine, University Center for Dental Medicine Basel UZB, Basel, Switzerland; 5 Shanghai Perio-Implant Innovation Center, Department of Oral and Maxillofacial Implantology, National Clinical Research Center of Stomatology, Ninth People’s Hospital, Shanghai Jiao Tong University School of Medicine, Shanghai, China

**Keywords:** ultrasonography, furcation defects, molar teeth, cone-beam computed tomography

## Abstract

**Objectives:**

The objectives were to clarify if intraoral ultrasonography (USG) is: (1) more accurate than conventional periodontal examinations in detection of furcation involvement, and (2) comparable to conventional periodontal examinations in accurate horizontal classification of furcation involvement in comparison to cone beam computed tomography (CBCT).

**Methods:**

The buccal furcation in 61 lower first molars were evaluated with conventional periodontal examinations, intraoral USG and CBCT. The presence and classification of the horizontal depth of furcation involvement were defined clinically by assessment with a Nabers periodontal probe and a periapical radiograph with reference to the bone loss under the fornix. The horizontal depth of furcation involvement was measured in intraoral USG and CBCT images. Based on the measurements, presence diagnosis and horizontal classification were performed. Results from conventional periodontal examinationsand intraoral USG were compared with those from CBCT.

**Results:**

κ value (κ) for agreement of presence diagnosis of furcation involvement between intraoral USG and CBCT was 0.792, while agreement with conventional periodontal examinations was 0.225. Diagnostic accuracy of intraoral USG exhibited higher values (sensitivity: 98.3%, accuracy: 98.4 %) than conventional periodontal examinations (81.4% and 81.9 %). Weighted κ statistics showed substantial agreement in the classification between intraoral USG and CBCT (κ = 0.674). High agreement (ICC: 0.914) for the measurement of horizontal depth of furcation involvement was found between intraoral USG and CBCT.

**Conclusions:**

Intraoral USG may be a reliable diagnostic tool for assessment of furcation involvement of mandibular molars with a similar performance to CBCT, but without ionizing radiation.

## Introduction

Molars, being multiple rooted teeth, are regarded as the most susceptible to periodontitis and are likely to develop furcation involvement.^1^ Furcation involvement is very common in periodontitis cases,^2^ and the class of furcation affects the longevity of the involved tooth.^3,4^ According to Hamp et al,^1^ furcation involvement can be classified into three classes by the extent of horizontal loss of the periodontal tissues: Class I, horizontal loss of periodontal tissue support less than 3 mm; Class II, horizontal loss of support 3 mm, but not encompassing the total width of the furcation; and Class III, horizontal through-and-through destruction of the periodontal tissue in the furcation. With the current periodontal disease classification, the presence of Class II or Class III furcation increases treatment complexity and, thus, also the stage of periodontal disease.^5^ Therefore, early detection and correct classification/diagnosis are of paramount importance. However, accurate measurement of the degree of furcation is challenging for the dentist in clinical practice.^6^ Clinical evaluation of furcation involvement is based on a combination of periodontal furcation probing with conventional two-dimensional (2D) intraoral radiographs.^6–16^ Unfortunately, periodontal furcation probing may not always be feasible, *e.g.* poor accessibility due to the morphology of furcation.^6,8,9,14,15^ Conventional 2D radiography is the current standard diagnostic procedure for the evaluation of periodontal bone tissue. However, there is a significant limitation in detecting bone defects on the facial and palatal/lingual surfaces of the affected teeth due to the projection of a 3D structure onto a 2D image.^7,8,11–16^

Previous studies revealed the usefulness of cone beam computed tomography (CBCT) for assessing furcation involvement.^11–17^ They concluded that CBCT images provide a highly accurate assessment of maxillary and/or mandibular molar furcation involvement.^11–16^ It has also been stated that the assessment by CBCT was comparable to that of direct surgical measurements.^15^ Meanwhile, over and above the general disadvantages of CBCT, such as limited soft tissue contrast, higher cost, higher radiation exposure, and artifacts around metal objects (crowns, bridges, implants, etc.), long-term hazards of effective dose accumulation limit its clinical application for routine monitoring of periodontal disease.^18^

All the above seem to justify the investigation of alternative diagnostic approaches, such as ultrasonography (USG), that could replace conventional 2D radiographs and/or CBCT in the assessment offurcation involvement. USG is an imaging technique based on the application of ultrasound with several advantages of being nonionizing, non-invasive, non-irritating, and a real-time imaging procedure.^19–22^ It can be used for both hard and soft tissue detection with unprecedented soft tissue contrast and well-delineated hard tissue surface without being affected by metal artifacts.^19–22^ Review articles^20–22^ suggested the clinical usefulness of intraoral USG in evaluating oral lesions such as tongue carcinomas, submandibular duct sialoliths and periapical lesions, as well as extraoral USG for the detection of lymph node metastases, salivary gland diseases, and temporomandibular joint disorders. Advanced intraoral ultrasound devices with high-frequency and small-footprint USG probes expanded the scope of intraoral application to the evaluation of teeth, as well as periodontal, and peri-implant pathologies.^23–33^ Numerous studies tried to recognize tooth-related periodontal and/or other anatomical structures on ultrasound images,^24–28,30^ assess the feasibility of measurements of crestal bone defects including peri-implant bone level and/or periodontal destruction,^23–25,29–31^ and evaluate its reliability as a diagnostic imaging tool for periodontal/peri-implant assessment.^25–27,29–31^ If intraoral USG could evaluate periodontal bone defects including furcation involvement accurately, USG probes could replace periodontal probes and 2D radiographs for monitoring periodontal conditions.

This study aimed to evaluate the practical usefulness and effectiveness of intraoral USG imaging as a clinical diagnostic tool for the detectability of the horizontal component of furcation involvement of lower first molars. The objectives of this study were to clarify: (1) if intraoral USG is more accurate than conventional periodontal examinations including periodontal probing and periapical radiographs in the detection of furcation involvement when compared with CBCT as the gold-standard (presence diagnosis), (2) if intraoral USG is comparable to conventional periodontal examinations in the accurate horizontal classification of horizontal depth of furcation involvement when compared with CBCT as the gold-standard (qualitative/quantitative diagnosis). To our best knowledge, no clinical study has yet reported whether intraoral USG is able to detect the presence and diagnose furcation involvement qualitatively/ quantitatively.

## Methods and patients

### Population under investigation

Thirty-five patients (20 females and 15 males) were randomly invited to participate in this study from patients for treatment of periodontitis or other dental purposes at Prince Philip Dental Hospital (PPDH) in Hong Kong. All the study participants had CBCT data of the lower first molars available that had been previously taken for the assessment of the periodontal condition or were scheduled for a CBCT for the treatment planning aside from the purpose of this study. All the subjects signed informed consent and underwent clinical, radiographical and intraoral USG examination in Prince Phillip Dental Hospital. The Institutional Review Board (IRB) of the University of Hong Kong approved the protocol of the study (UW-20–304).

Subject Inclusion criteria:

- Dental patients having at least one first lower molar with/without furcation involvement
- Dental patients who had a series of images from a CBCT scan including the lower first molar obtained less than 6 months before/after the clinical and intraoral USG evaluation

Subject exclusion criteria:

- Dental patients who were younger than 18 years old
- Dental patients who had undergone any periodontal surgery on the examined molars
- Dental patients who had severe acute inflammatory conditions around the examined molar, such as persisting bleeding, ulcer, and/or severe pain
- Dental patients with gross amounts of calcified deposits supra- or subgingivally.
- Dental patients undergoing orthodontic treatment with dental appliance
- Dental patients with any syndromes that cause the tooth malformation

### Conventional periodontal examinations

All the lower first molars from 35 patients were assessed with a standardized periodontal examination by periodontal probing as well as periapical radiographs.

The presence of furcation involvement and degree of horizontal depth of furcation involvement was defined clinically by inserting a Nabers periodontal probe and confirmed in combination with a periapical radiograph with reference to bone loss under the fornix. Conventional periodontal examinations of furcation involvement including periodontal probing and intraoral periapical radiographs were performed by one practitioner (KL). The presence of furcation involvement was assessed using the buccal periodontal probing data and periapical radiographs with a certified periodontist (GP). The presence and classification of horizontal depth of furcation involvement were determined by the finding exhibiting the most advanced interradicular bone defect in the horizontal plane. For cases in which a discrepancy was found between periodontal probing and radiographic examination, a certified periodontist (GP) was consulted.

The classification was performed as follows^1^:

Class 0: No horizontal loss of periodontal tissue

Class I: Horizontal loss of periodontal tissue support less than 3 mm.

Class II: Horizontal loss of support 3 mm, but not encompassing the total width of the furcation.

Class III: Horizontal through-and-through destruction of the periodontal tissue in the furcation.

### Intraoral USG examination

**Figure 1.**
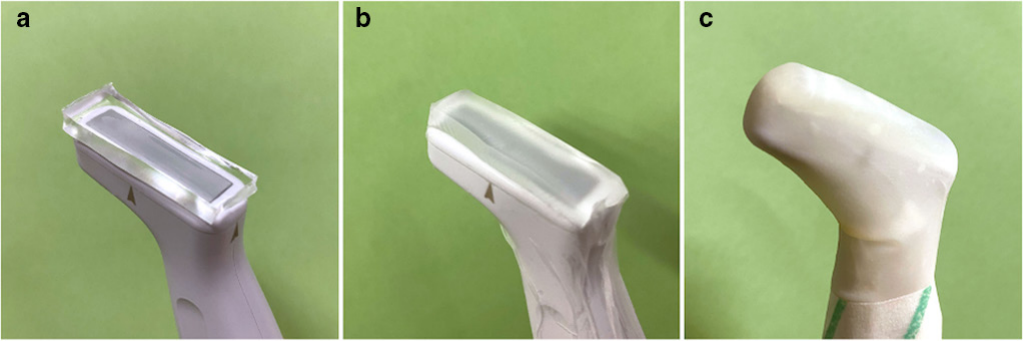
USG probe preparation. (**A**) The acoustic coupling agent on the USG probe. (**B**) Covered with waterproof bandage. (**C**) Covered with a latex bag. USG, ultrasonography

Intraoral USG examination was performed for all lower first molars by a single experienced maxillofacial radiologist (RT) blinded with respect to the clinical findings and CBCT results. A commercially available ultrasound diagnostic system (SONIMAGE HS1, Konica Minolta Ltd. Tokyo, Japan) with a wideband and high-frequency hockey stick linear USG probe (SONIMAGE HS1 HL18-4) was used for B-mode imaging. Before intraoral USG examination, mouth gel was applied to the experimental area to keep away the air potentially causing artifacts. The high molecular acoustic coupling agent was cut in size to be placed on the USG probe. The USG probe with the acoustic coupling agent was covered with a waterproof bandage and latex bag (Figure 1). The USG probe was placed on the buccal surface of the examined tooth and/or surrounding structures to obtain axial and/or para-coronal sections against the long axis of the tooth. The image data in cine and static modes were acquired.

**Figure 2.**
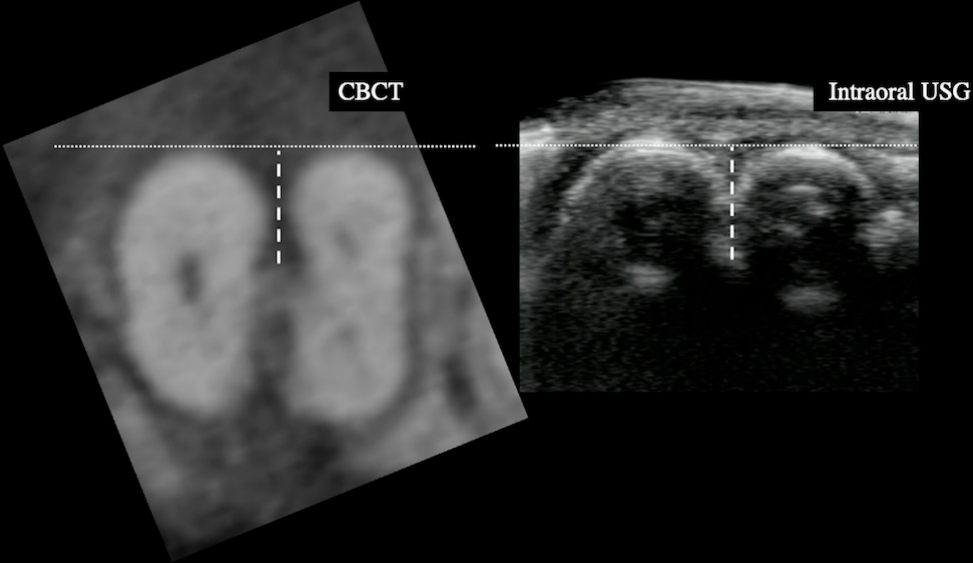
Measurement of the horizontal depth of furcation involvement on CBCT and intraoral USG images. Measurement was performed from the buccal surface of the roots to the end of the bone defect. The line with larger dots shows the measurement. CBCT, cone beam CT; USG, ultrasonography.

**Figure 3.**
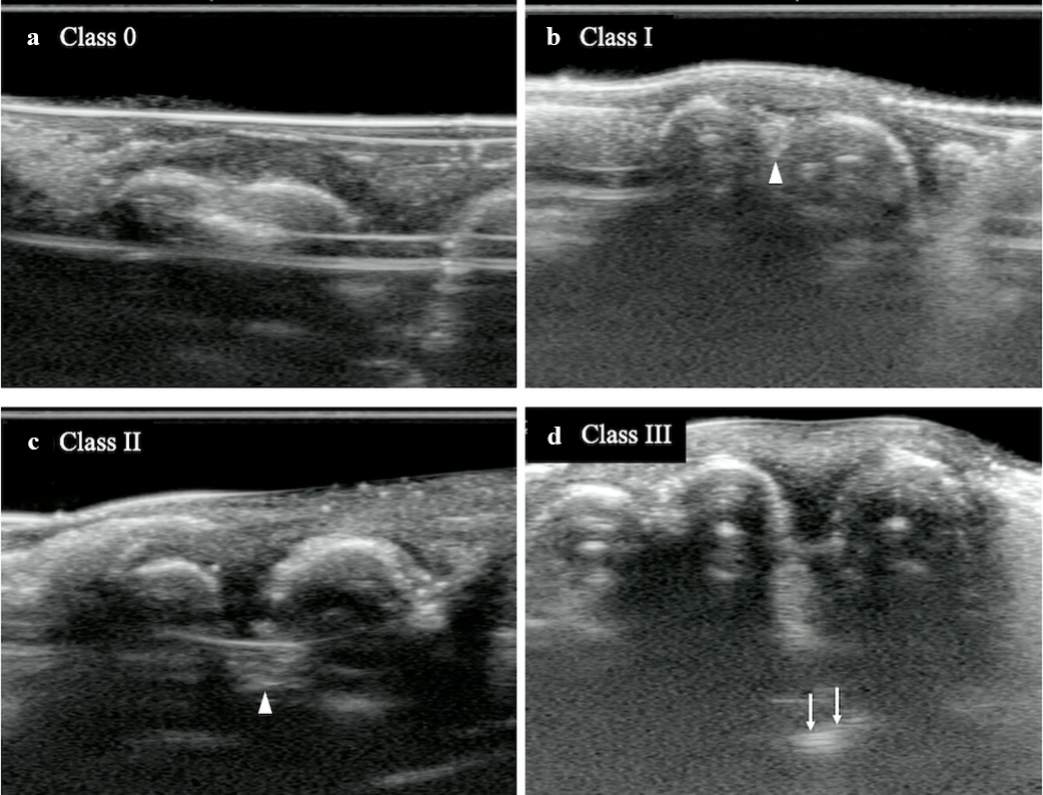
Classification of furcation involvement in B-mode intraoral USG. (**A**) Axial (Cross-sectional) image of a case without furcation involvement (Class 0). Interradicular part is not indicated. (**B**) Axial (Cross-sectional) image of a case classified in Class I. A V-shaped bone defect is observed between the roots. A white arrowhead indicates the end of bone defect. (**C**) Axial (Cross-sectional) image of a case classified in Class II. Considerably deep bone defect is demonstrated. A white arrowhead indicates the end of bone defect. (**D**) Axial (Cross-sectional) image of a case classified in Class III. Hyperechoic line from tongue mucosa (white arrows) is suspected at the end of interradicular bone defect. USG, ultrasonography.

Intraoral USG image data of 61 lower first molars were included for the image analysis. All image data were saved in DICOM format, anonymized, and randomized for analysis using an image processing software (OsiriX MD v. 8.0.2 for Mac, Pixmeo, Switzerland) on the high-resolution display. The presence of furcation involvementin the intraoral USG imaging was first determined, followed by detecting the deepest horizontal bone defect in the furcation and measuring the distance between the buccal surface of the roots and the deepest point of interradicular bone loss as the horizontal depth of furcation involvement on the axial images in cine (Figure 2). The classification mentioned above was applied to every furcation involvement according to the result of the measurements (Figure 3). The assessment was performed twice, and the second was performed more than 1 month after the first. Any disagreement of the diagnosis between the first and the second assessment was discussed with another oral and maxillofacial radiologist (TH).

### CBCT examination

The CBCT scans including the lower first molars had been taken with two CBCT systems (Planmeca ProMax 3D Mid, Planmeca, Helsinki, Finland/ X800, J. Morita, Kyoto, Japan) at Prince Philip Dental Hospital. The scanning protocol varied between field of view sizes of 80 × 80 mm, 100 × 100 mm, or 200 × 170 mm, and voxel sizes of 0.2 or 0.4 mm.

CBCT data taken within 6 months from conventional periodontal examinations and intraoral USG were saved in DICOM format. A total of 61 lower first molars (29 were scanned before, 32 after periodontal examination and intraoral USG) were included for the image analysis. Anonymization and randomization of the DICOM files were performed. The CBCT data were evaluated using a medical image processing software (OsiriX MD v. 8.0.2 for Mac, Pixmeo, Switzerland) on the high-resolution display—the same as for intraoralUSG data analysis. The presence or absence of furcation involvement in the CBCT imaging was determined. The deepest horizontal bone defect in the furcation was detected on the multiplanar reconstruction (MPR) images and the distance between the buccal surface of the roots and the deepest point of interradicular bone loss was measured as the horizontal depth of furcation involvement (Figure 2).

The furcation involvement was classified into Class I, II, and III^1^ according to the linear measurement of the horizontal depth of furcation involvement on the axial images. One oral and maxillofacial radiologist (AY) performed the assessment twice with no information on the results of intraoral USG and conventional periodontal examinations. The second assessment was performed more than 1 month after the first. In case of disagreements in diagnosis between the first and the second assessment, this was discussed with a certified periodontist (WKL).

### Statistical analysis

The sample size was calculated using G*Power v. 3.1.9.2 (Franz Faul, University of Kiel, Germany) based on a previous study conducted by Zhang et al.^14^ The power analysis demonstrated that a total sample size of 17 subjects would achieve 80% power to detect the relation between the three diagnostic methods on a significant level of 0.05.

All the results from conventional periodontal examinations and intraoral USG were compared to the data from CBCT as the gold-standard in this study. Accuracy, sensitivity and specificity were calculated for assessment of the ability to diagnose furcation involvement with conventional periodontal examinations and intraoral USG. Cohen’s κ test was used to show the agreement for the presence of furcation involvement between conventional periodontal examinations, intraoral USG, and CBCT. Cohen’s weighted κ coefficient was calculated to assess the agreement of the classification of horizontal depth offurcation involvement among the three methods. Intraclass correlation coefficient and Bland–Altman analysis were applied for linear measurement values from CBCT and intraoral USG.

SPSS v. 28 (IBM Corp. Armonk, NY) was used for all statistical analyses. Results with *p*-values of <0.05 were considered statistically significant. For the interpretation of Cohen’s κ values, the following grading was used: <0: No agreement, 0–0.20: Slight, 0.21–0.40: Fair, 0.41–0.60: Moderate, 0.61–0.80: Substantial, 0.81–1.0: Perfect. For the interpretation of the ICC values, the following grading was used: ≤0.75: moderate to poor, 0.75–0.90: good, ≥0.90: high.

## Results

Buccal furcations of 61 lower first molars (29 on the left and 32 on the right) in 35 patients (15 males and 20 females) were evaluated using conventional periodontal examinations, intraoral USG and CBCT imaging. Results from conventional periodontal examinations and intraoral USG were compared with CBCT as the gold-standard in this study. The results of the diagnosis for the presence of furcation involvement in conventional periodontal examinations and intraoral USG are demonstrated in Table 1. The present study showed a 19.7% absence of furcation involvement in conventional periodontal examinations compared to 3.2% in CBCT. Cohen’s κ value for agreement of the diagnosis for furcation involvement of intraoral USG with CBCT was interpreted as nearly perfect agreement (κ = 0.792), while that of conventional periodontal examinations was fair (κ = 0.222) (Table 1).

**Table 1.** Agreement of diagnosis for furcation involvement comparing conventional periodontal examinations and intraoral USG with CBCT

		CBCT			
		Presence	Absence	κ	Sig.
**Conventional periodontal examinations**	Presence	48	0	0.222	0.006
	Absence	11	2		
**Intraoral** **USG**	Presence	58	0	0.792	<0.001
	Absence	1	2		
Total		59	2		

CBCT, cone beam CT; USG, ultrasonography.

Sensitivity, specificity and accuracy in diagnostic methods of conventional periodontal examinations and intraoral USG were calculated. Intraoral USG exhibited higher values (sensitivity: 98.3%, specificity: 100%, accuracy: 98.4%) than conventional periodontal examinations (81.4%, 100%, and 81.9%).

**Figure 4.**
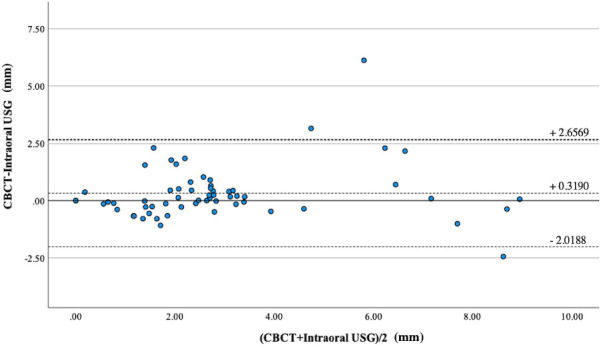
Bland–Altman plot for comparison of linear measurements with intraoral USG with CBCT. According to Bland–Altman Analysis, it was found that intraoral USG had a smaller measurement of furcation involvement than CBCT (fixed error). There was no significant correlation (proportional error) between two valuables. CBCT, cone beam CT; USG, ultrasonography.

Regarding horizontal classification of furcation involvement, Cohen’s weighted κ statistic showed substantial agreement in the classification of furcation involvement between intraoral USG and CBCT (κ = 0.691, 95% confidence interval = 0.539–0.844, *p* < 0.001). In contrast, a moderate agreement was found between conventional periodontal examinations and CBCT (κ = 0.505, 95% confidence interval = 0.343–0.667, *p* < 0.001). Table 2 two revealed that conventional periodontal examinations underestimated more Class I and II cases than intraoral USG. Regarding the degree of classification, a higher agreement with CBCT for Class I (94.4 %), Class II (53.8 %), and Class III (50.0 %) was generally observed for intraoral USG in comparison to conventional periodontal examinations (Class I: 72.2%; Class II 30.8%; and Class III: 50.0 %). High agreement (ICC: 0.922) for the linear measurement of furcation involvement depth was found comparing intraoral USG and CBCT. According to Bland–Altman analysis (Figure 4), intraoral USG resulted in smaller measurements for furcation involvement than CBCT (fixed error). There was no significant correlation (proportional error) between the measurements of intraoral USG and CBCT.

**Table 2.** Cross-table of horizontal classification of horizontal depth of furcation involvement comparing conventional periodontal examinations and intraoral USG with CBCT

		CBCT				Total
		0	I	II	III	
**Conventional periodontal examinations**	0	2	10	0	0	12
		2	1	0	0	3
	I	0	26 (72.2 %)	9	2	37
		0	34 (94.4 %)	6	1	41
**Intraoral USG**	II	0	0	4 (30.8 %)	3	7
		0	1	7 (53.8 %)	4	12
	III	0	0	0	5 (50.0 %)	5
		0	0	0	5 (50.0 %)	5
Total		2	36	13	10	61
		2	36	13	10	61

CBCT, cone beam CT; USG, ultrasonography.

a (): Agreement degree (%) with CBCT in each class.

## Discussion

Currently, the clinical application of intraoral USG in dentistry is mainly limited to evaluating oral lesions, such as tongue carcinoma, intraoral mucosal disease, submandibular duct sialoliths and periapical lesions.^20–22^ In recent years, availability of high-resolution ultrasound devices and the small-footprint USG probes has shifted the focus of intraoral USG use towards teeth and periodontal/peri-implant tissues. Numerous pre-clinical and clinical studies have been published, providing new data concerning ultrasound imaging in the fields of periodontology and implantology.^32,33^ In the current study, the focus was on the diagnostic application of intraoral USG for furcation involvement assessment.

The results from this study revealed that intraoral USG is able to detect furcation involvement almost to perfection when compared with CBCT. Only one disagreement with CBCT findings in a total of 61 cases included here was seen. This was corroborated by the Cohen’s κ values for agreement of furcation involvement diagnosis comparing intraoral USG with CBCT. The results of this study demonstrated that intraoral USG yielded comparable results to CBCT imaging to detect the presence of furcation involvement. Additionally, the ability of intraoral USG to diagnose furcation involvement appeared to be superior to that of conventional periodontal examinations. Only one tooth was misdiagnosed, which showed a slight bone defect at the buccal entrance of the furcation at the fornix in the respective CBCT images. The actual buccal alveolar bone level was higher than the level of the furcation, with a narrow bone defect along the tooth root trunk to the furcation entrance. Successful detection of a furcation involvement using intraoral USG may also depend on operator-dependent and site-related factors, such as operators’ skill, USG probe head designs, or intraoral accessibility.^6,8,14,15^ The present study showed a 19.7% absence of furcation involvement in conventional periodontal examinations compared to 3.2% in CBCTs for mandibular buccal furcation involvement. Although the diagnostic performance of periodontal probing and periapical radiography were not separately analyzed, the present study results concerning the ability of furcation involvement diagnosis may be due to an underdetection of furcation involvement by periodontal probing, which had been reported similarly by several other groups.^10,12,14^ In the current study, five teeth in which a negative finding in the presence diagnosis was observed on periodontal probing were actually positive in 2D radiography, whereas one tooth was diagnosed with no furcation involvement in radiography, but furcation involvement was present upon periodontal probing. Such findings are consistent with the study by Zhang et al,^14^ where a higher diagnostic ability could be expected by combining the methods of periodontal probing and 2D imaging.

Intraoral USG exhibited a “substantial” agreement with CBCT for the classification of the horizontal component of furcation involvement in our study. Comparing each class, intraoral USG correctly diagnosed nearly all of the cases that CBCT determined as Class I, while conventional periodontal examinations correctly diagnosed two-thirds of all Class I cases. Contrary to this, intraoral USG was not very accurate in the diagnostic classification for Class II and Class III. Only half of all the cases of Class II and Class III were correctly diagnosed when compared to CBCT. Additionally, intraoral USG underestimated CBCT diagnosis, similar to the findings based on periodontal probing. Bland–Altman analysis of the actual value of linear measurements of horizontal depth of furcation involvement comparing intraoral USG and CBCT also indicated that intraoral USG generally resulted in smaller measurements compared to CBCT. The time gap between intraoral USG and CBCT examination might have had an effect on the result. Bertrum et al described that intraoral USG underestimated marginal bone loss in advanced bone loss cases when compared with surgical measurements.^23^ They mentioned possible reasons for this, such as the difficulty in orientating the USG probe and the angulation of the implant relative to the buccal bone. Operator experience, USG probe angulation, and/or its design may have affected the results in the present study. Besides, the deeper the bone defect, the more complicated the pathological condition might be found, such as an intricate morphology of the bony defect, or calcification in the defect area. In such cases, intraoral USG may have underestimated the extent of the defect due to the characteristics that the tissues/materials which strongly reflect the sound make it unable to evaluate the tissues behind them. Aside from that, the strength of intraoral USG in diagnosing Class I furcation involvement should be much valued as it might be a promising diagnostic tool for early detection of furcation involvement.

The current study had some limitations. First, the small-footprint hockey stick linear USG probe used could only reach the lingual furcation in a few cases. Therefore, the assessment was limited to the buccal furcation. Some cases were difficult even on the buccal sites due to a combination of the problems from site accessibility and configuration of the USG probe. The size, shape, and material of the USG probe should be reconsidered for successful use in the oral cavity. The second limitation was the difficulty of accurate measurement of through-and-through furcation involvement. Ultrasound imaging in the furcation area relies on the reflection of soft and hard tissues on the lingual aspect in order to determine Acknowledgments. In through-and-through furcation involvement, where there is no alveolar bone crest or gingiva on the lingual side, no reflection of ultrasound can be observed. As the opposite margin of the root cannot be observed, the lingual limit can only be determined by an echo of the tongue. Because of the limitations described above, a routine use of intraoral USG for furcation involvement assessment in daily practice is still debatable at this stage. Further studies focusing on innovations in ultrasound technology and refining furcation involvement assessment methodology are necessary to implement intraoral USG as a valuable diagnostic tool in periodontal practice. A third limitation was that CBCT data were used as a standard for comparison. Based on numerous previous studies that verified the diagnostic accuracy of CBCT in the assessment of bone defects, CBCT was used as the most reliable standard instead of intrasurgical measurement. According to a systematic review,^17^ however, the diagnostic accuracy of CBCT is not perfect when compared to the result from direct measurement. Qiao et al reported that CBCT not only underestimated but also overestimated furcation bone defects relative to the respective intrasurgical measurements.^12^ Meanwhile, Warda et al^11^ and Padmanabhan et al^15^ observed no significant difference between CBCT and intrasurgical measurements.

## Conclusions

USG has become more popular as a novel imaging modality in dentistry, especially in periodontology and implantology. The future development of various USG probe head designs in size, shape and/or materials could help practitioners to assess teeth and periodontium more easily and accurately and enable this real-time and non-ionizing imaging technique to be a diagnostic tool for the dental office. The results of the current study demonstrated that intraoral USG was comparable to: (1) CBCT imaging in presence diagnosis of furcation involvement in mandibular first molars, (2) CBCT imaging in the horizontal classification of furcation involvement of Class I. Thus, intraoral USG may be a reliable diagnostic tool for furcation involvement of mandibular molars.
